# Treatment patterns among children and adolescents with attention-deficit/hyperactivity disorder in the United States – a retrospective claims analysis

**DOI:** 10.1186/s12888-022-04188-4

**Published:** 2022-08-18

**Authors:** Jeff Schein, Ann Childress, Julie Adams, Patrick Gagnon-Sanschagrin, Jessica Maitland, Wendi Qu, Martin Cloutier, Annie Guérin

**Affiliations:** 1 Otsuka Pharmaceutical Development & Commercialization, Inc., 508 Carnegie Center Dr, Princeton, NJ 08540 USA; 2 Center for Psychiatry and Behavioral Medicine, Inc., 7351 Prairie Falcon Rd STE 160, Las Vegas, NV 89128 USA; 3 Analysis Group, Inc., 1190 avenue des Canadiens-de-Montréal, Tour Deloitte, Suite 1500, Montreal, QC H3B 4W5 Canada

**Keywords:** Attention deficit hyperactivity disorder, ADHD, Treatment pattern, Discontinuation, Switch, Child, Adolescent, Costs

## Abstract

**Background:**

Attention-deficit/hyperactivity disorder (ADHD) is a common neurobehavioral disorder affecting approximately 10.0% of children and 6.5% of adolescents in the United States (US). A comprehensive assessment of the current treatment landscape is warranted to highlight potential unmet needs of children and adolescents with ADHD. Therefore, this study described treatment patterns and healthcare costs among commercially insured children and adolescents with ADHD in the US.

**Methods:**

Children and adolescents with ADHD initiating pharmacological treatment indicated for ADHD were identified from IBM MarketScan Commercial Database (2014–2018). A treatment sequence algorithm was used to examine treatment patterns, including discontinuation (≥ 180 days following the last day of supply of any ADHD treatment), switch, add-on, and drop (discontinuation of an agent in combination therapy), during the 12-month study period following the index date (i.e., first observed ADHD treatment). Total adjusted annual healthcare costs were compared between patients with and without treatment changes.

**Results:**

Among 49,756 children and 29,093 adolescents included, mean age was 9 and 15 years, respectively, and 31% and 38% were female. As the first treatment regimen observed, 92% of both children and adolescents initiated a stimulant and 11% initiated combination therapy. Over half of the population had a treatment change over 12 months—59% of children and 68% of adolescents. Treatment discontinuation over 12 months was common in both populations—21% of children and 36% of adolescents discontinued treatment. Healthcare costs increased with the number of treatment changes observed; children and adolescents with treatment changes (i.e., 1, 2, or ≥ 3) incurred an incremental annual cost of up to $1,443 and $2,705, respectively, compared to those without a treatment change (*p* < 0.001). Costs were largely driven by outpatient visits.

**Conclusions:**

Over a 12-month period, treatment changes were commonly observed and were associated with excess costs, highlighting the unmet treatment needs of children and adolescents with ADHD in the US.

## Background

Attention-deficit/hyperactivity disorder (ADHD) is a common neurobehavioral disorder affecting approximately 10.0% of children and 6.5% of adolescents in the United States (US) [1–3]. ADHD presentation may be predominantly inattentive (e.g., disorganized and forgetful), predominantly hyperactive-impulsive (e.g., extreme restlessness and tendency to interrupt others), or combined (i.e., criteria for both inattentive and hyperactive-impulsive ADHD are met) [4]. The manifestation of ADHD may impose a significant educational and psychosocial impact on children and adolescents with the condition [5–7], resulting in a high patient burden that commonly extends to their caregivers [8, 9]. Additionally, persistent ADHD symptoms may have long-term consequences, such as difficulty in securing stable employment and impaired productivity [10]. A recent study showed that 91% of patients with childhood ADHD do not achieve sustained remission into adulthood [11], suggesting lasting effects of ADHD across the patients’ life course.

Management options for children and adolescents with ADHD include non-pharmacological approaches, such as psychotherapy (e.g., behavioral parental and classroom training) and organizational training (e.g., target skill development), as well as pharmacological treatment (e.g., with short- and long-acting stimulants or non-stimulants) [3]. In the US, approximately two-thirds of children and adolescents diagnosed with ADHD are receiving ADHD indicated medications [12] that have been shown to reduce ADHD symptoms [13]. However, among these patients treated with pharmacological options, treatment adherence and persistence tend to be low [14, 15]. ADHD treatments have also been associated with adverse effects such as appetite loss, sleep disturbances, and growth delays [3, 16] that may cause some parents of children and adolescents with ADHD to prefer psychotherapy options [17], which often requires heavy family or school involvement and may result in strains to family members and teachers [3]. Suboptimal management of ADHD among children and adolescents may lead to poorly controlled symptoms and lead to an increased burden on the patients as well as their parents, teachers, and peers.

To assess if the current treatment landscape meets the needs of children and adolescents with ADHD, an evaluation of ADHD treatment patterns (such as discontinuation and switching) among these populations is warranted, as frequent treatment changes may suggest suboptimal management with current treatments. Previous studies on ADHD treatment patterns have generally focused on specific subgroups of interest (e.g., a particular drug/drug class [18–20] or patient population [21]), and few studies have evaluated treatment changes in children and adolescents separately using the same methodologies to allow for the identification of any population-specific discrepancies. Importantly, limited information exists on the sequences of use of various ADHD medications in cases of multiple treatment changes that can provide insight to the journeys of patients with ADHD.

Furthermore, treatment changes can be costly, as evidenced in mental health conditions other than ADHD [22, 23]. For instance, treatment discontinuation may lead to additional clinic visits due to uncontrolled symptoms, and treatment switch may result in increased use of acute healthcare services due to adverse effects of new treatments [22]. Thus, healthcare costs associated with treatment changes may represent an undue burden, but this has not been extensively studied in ADHD. Therefore, the current study sought to comprehensively assess treatment patterns and the associated healthcare costs among children and adolescents with ADHD in a real-world setting. Findings of this study may inform clinicians and policymakers of the potential unmet need in existing clinical management of ADHD in these patient populations and help raise awareness of the associated burden.

## Methods

### Data source

Data from the IBM MarketScan Commercial Database covering the period between 2014 and 2018 were used. The database covers over 200 million individuals from more than 120 contributing employers and 40 contributing health plans. The database consists of medical and drug data for employees, their spouses, and dependents covered by employer-sponsored private health insurance and includes records of inpatient services, inpatient admissions, outpatient services, and prescription drug claims [24]. Data are de-identified and comply with the patient requirements of the Health Insurance Portability and Accountability Act. Therefore, no institutional review board exemption was required.

### Study design and populations

A retrospective claims-based analysis was conducted to assess treatment patterns among two mutually exclusive cohorts of patients with ADHD—children (aged 6–12 years) and adolescents (aged 13–17 years)—who initiated a Food and Drug Administration (FDA)–approved pharmacological treatment for ADHD (i.e., stimulants, non-stimulants) following a 6-month period without receiving any ADHD treatment to capture a newly initiated ADHD treatment regimen. The study period was defined as the 12-month period following the first observed ADHD prescription fill date (i.e., index date) and the follow-up period was defined as the 6-month period following the study period (i.e., patients were required to have 18 months of continuous health plan enrollment following the index date). The additional 6 months of follow up allowed for sufficient time to determine treatment changes within the entirety of the 12-month study period. The baseline period was defined as the 6-month period preceding the index date. The study design allowed for information on treatments received to be captured among a representative population of children and adolescents with ADHD, including both patients newly initiating a first-line treatment and previously treated patients starting a new treatment regimen.

Children and adolescents were included in the study if they met the following criteria: 1) had ≥ 2 diagnoses of ADHD on distinct dates at any time in the data (International Classification of Diseases, Ninth/Tenth revision, Clinical Modification [ICD-9-CM codes: 314.0x; ICD-10-CM codes: F90.x]); 2) had ≥ 1 prescription fill for an FDA-approved ADHD treatment on or after the first observed ADHD diagnosis; 3) had continuous health plan enrollment ≥ 6 months prior to and 18 months following the index date; and 4) had a 6-month washout period prior to the index date in which there was no prescription fill for an ADHD-related treatment. Patient characteristics were compared before and after applying criteria number 3 to ensure generalizability was maintained.

Patients who did not have a treatment change (defined in the next section) observed during the 12-month study period (i.e., patients who remained on the first ADHD-related agent(s) observed in the data following the ADHD diagnosis) were classified into the no treatment change cohort and patients who had ≥ 1 treatment change observed during the 12-month study period were classified into the treatment change cohort. The treatment change cohort was further stratified into 3 mutually exclusive cohorts based on the number of treatment changes observed (i.e., 1, 2, or ≥ 3).

### Treatment changes and sequences

**Fig. 1:**
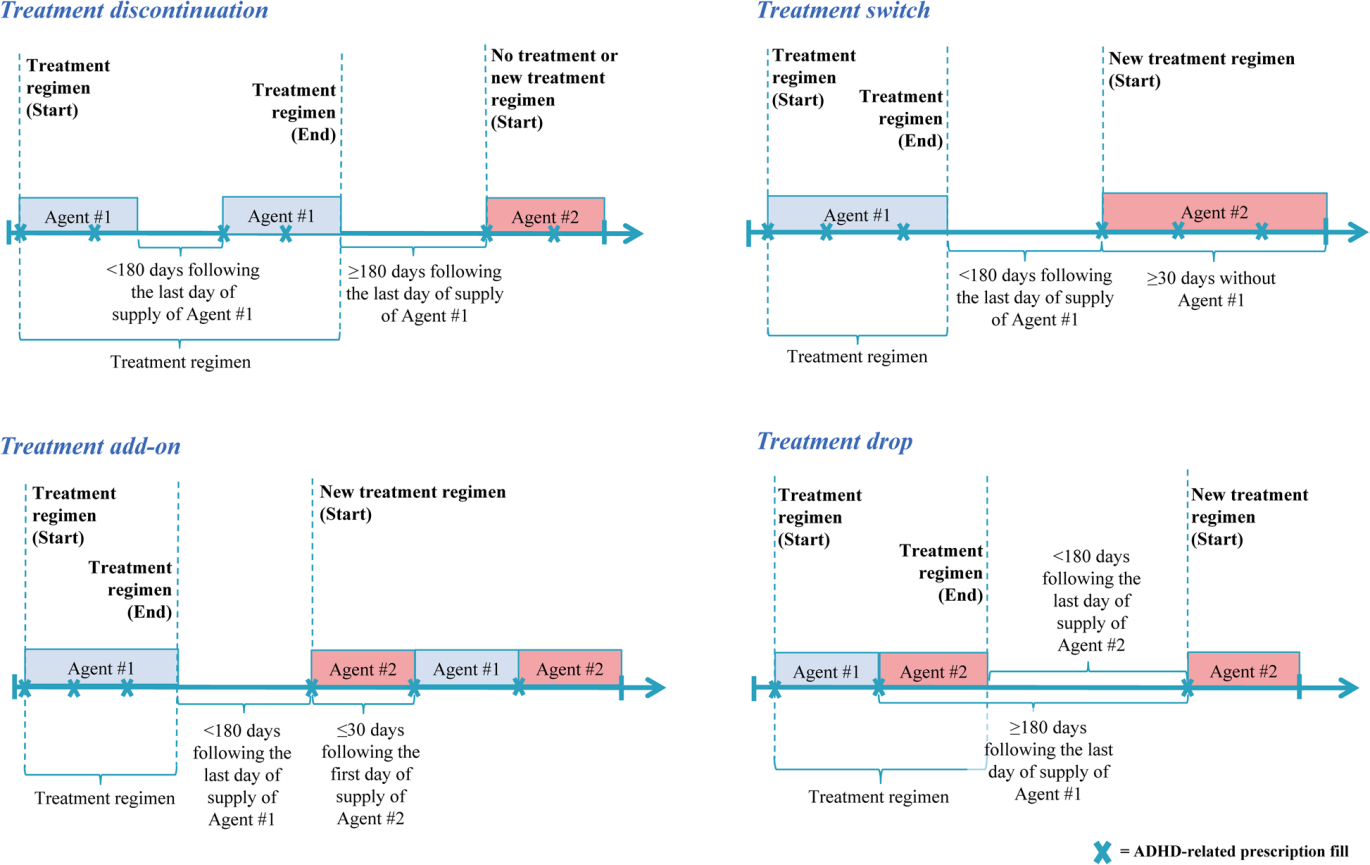
Schematic of treatment change definition

Treatment changes and sequences were examined at the agent level during the 12-month study period using a specific algorithm [25]. Agents with the same active ingredient were considered as the same medication, regardless of dosage or brand. Dose changes and dose titration were not considered as a treatment change and were included in the same treatment regimen. Treatment sequences of up to 3 consecutive treatment regimens were described. A treatment regimen was defined as all ADHD-related agents observed within 30 days of the first ADHD-related agent, wherein the first ADHD-related agent was identified using all prescription fills observed during the 12-month study period. For example, if a patient received agent X and then 15 days later received agent Y, the treatment regimen for the patient would be a combination therapy of agent X plus agent Y (Fig. 1). The start of a treatment regimen was defined as the date of the first prescription fill of an ADHD-related agent and the end of a treatment regimen was defined as the first ADHD-related treatment change or the end of the 12-month study period, whichever occurred earlier.

Treatment changes observed included treatment discontinuation (defined as no ADHD-related agents for ≥ 180 consecutive days following the last day of supply of the treatment regimen), treatment switch (defined as initiation of a new ADHD-related agent with no prescription fills from the previous treatment regimen within the 30 days following initiation), treatment add-on (defined as initiation of a new ADHD-related agent with ≥ 1 other prescription fill from the previous treatment regimen within the 30 days following initiation), and treatment drop (defined as the discontinuation of an ADHD-related agent from a combination regimen while the other agent[s] from the regimen are not discontinued; Fig. 1). Requiring ≥ 180 days following the last day of supply of any ADHD treatment to be considered a treatment discontinuation or a treatment drop allowed for drug holidays and treatment interruptions to be accounted for. To investigate how results may differ by class of ADHD medication, treatment changes were also described among a subgroup of patients whose first treatment regimen observed consisted of only stimulants and those with only non-stimulants, in either monotherapy or combination therapy of multiple agents within the same class. Treatment combinations (i.e., ≥ 2 ADHD-related agents in a regimen) and non-pharmacological treatments (i.e., psychotherapy) were also descriptively reported for the first 3 treatment regimens observed.

### Study measures and outcomes

The study measures and outcomes included baseline patient characteristics (e.g., age, gender, type of ADHD diagnosis [diagnosis of combined ADHD was only available from October 2015], comorbidities, prior pharmacological treatment), treatment characteristics (e.g., types of pharmacological treatment at the class and agent levels, receipt of treatment combinations and/or psychotherapy, and treatment duration of the first 3 regimens observed), treatment changes, and total annual healthcare costs associated with treatment changes. Total annual healthcare costs comprised medical (i.e., inpatient, outpatient, and emergency department) and pharmacy costs and were measured from the index date until the end of the 12-month study period. Total annual healthcare costs among patients with and without a treatment change, as well as that among subgroups with 1, 2, or ≥ 3 treatment changes were compared. Costs were assessed from the payers’ perspective and adjusted to 2019 US Dollar using the medical care component of the US Consumer Price Index [26]. All outcomes were reported separately for the children and adolescents with ADHD.

### Statistical analysis

Baseline patient characteristics, treatment characteristics, and treatment changes were reported using means, standard deviations, and medians for continuous variables and frequencies counts and percentages for categorical variables. Differences in the total annual healthcare costs were compared using ordinary least squares regression models with robust standard errors. Adjusted annual healthcare cost differences were reported along with their 95% confidence intervals (CI), using robust standard errors, and p-values. The following control variables were used in the regression for cost differences (including a priori selected demographic characteristics and clinical characteristics with a standardized difference ≥ 0.1 between cohorts): age, gender, type of health plan, region of residence, year of index date, type of ADHD, anxiety (children only), psychotherapy visits during the study period (children only), and depression (adolescents only).

## Results

### Baseline patient characteristics

A total of 49,756 children with ADHD who met the study criteria were included. Among children with ADHD, the mean age was 8.8 years and 30.8% were female; these demographics were similar to that of the sample prior to requiring continuous eligibility. The first observed ADHD diagnosis among children was commonly hyperactive ADHD (43.9%), followed by inattentive ADHD (30.8%), and combined type ADHD (17.1%). During the 6-month baseline period, 11.1% of children with ADHD had depression and 11.0% had anxiety disorders. Other common baseline comorbidities included acute upper respiratory infections (26.3%) and acute pharyngitis (15.5%). Frequently used pharmacological treatments during the baseline period among children with ADHD included penicillins (17.0%) and antiasthmatic and bronchodilator agents (12.0%) (Table 1).

**Table 1:** Characteristics of children and adolescents with ADHD – Overall and by treatment change cohort

	**Children**				**Adolescents**			
**Patient characteristics**^a^	**All patients**	**No treatment change cohort**	**Treatment change cohort**	**Standardized difference**^b^	**All patients**	**No treatment change cohort**	**Treatment change cohort**	**Standardized difference**^b^
	***N* = 49,756**	***N* = 20,375**	***N* = 29,381**		***N* = 29,093**	***N* = 9,377**	***N* = 19,716**	
**Demographic characteristics**^**c**^								
**Age, mean ± SD [median]**	8.75 ± 1.92 [9.00]	8.76 ± 1.87 [9.00]	8.74 ± 1.94 [9.00]	0.01	15.02 ± 1.37 [15.00]	14.93 ± 1.37 [15.00]	15.06 ± 1.37 [15.00]	0.10
**Female, N (%)**	15,332 (30.8)	6,215 (30.5)	9,117 (31.0)	0.01	11,078 (38.1)	3,494 (37.3)	7,584 (38.5)	0.02
**Health plan type, N (%)**								
Preferred provider organization	28,267 (56.8)	11,646 (57.2)	16,621 (56.6)	0.01	16,328 (56.1)	5,351 (57.1)	10,977 (55.7)	0.03
Consumer driven health plan	7,095 (14.3)	2,978 (14.6)	4,117 (14.0)	0.02	4,063 (14.0)	1,355 (14.5)	2,708 (13.7)	0.02
Home maintenance organization	5,577 (11.2)	2,278 (11.2)	3,299 (11.2)	0.00	3,304 (11.4)	1,028 (11.0)	2,276 (11.5)	0.02
High deductible health plan	4,304 (8.7)	1,598 (7.8)	2,706 (9.2)	0.05	2,580 (8.9)	781 (8.3)	1,799 (9.1)	0.03
Point of service, no capitation	2,790 (5.6)	1,191 (5.8)	1,599 (5.4)	0.02	1,667 (5.7)	522 (5.6)	1,145 (5.8)	0.01
Comprehensive	1,096 (2.2)	424 (2.1)	672 (2.3)	0.01	777 (2.7)	231 (2.5)	546 (2.8)	0.02
Exclusive provider organization	349 (0.7)	137 (0.7)	212 (0.7)	0.01	232 (0.8)	66 (0.7)	166 (0.8)	0.02
Point of service, partially or fully capitated	181 (0.4)	78 (0.4)	103 (0.4)	0.01	91 (0.3)	33 (0.4)	58 (0.3)	0.01
Unknown	97 (0.2)	45 (0.2)	52 (0.2)	0.01	51 (0.2)	10 (0.1)	41 (0.2)	0.03
**Region of residence, N (%)**								
South	26,036 (52.3)	11,160 (54.8)	14,876 (50.6)	0.08	26,036 (52.3)	4,698 (50.1)	9,333 (47.3)	0.06
North central	10,753 (21.6)	4,382 (21.5)	6,371 (21.7)	0.00	10,753 (21.6)	2,205 (23.5)	4,527 (23.0)	0.01
West	6,911 (13.9)	2,622 (12.9)	4,289 (14.6)	0.05	6,911 (13.9)	1,314 (14.0)	2,964 (15.0)	0.03
Northeast	5,984 (12.0)	2,182 (10.7)	3,802 (12.9)	0.07	5,984 (12.0)	1,140 (12.2)	2,858 (14.5)	0.07
Unknown	72 (0.1)	29 (0.1)	43 (0.1)	0.00	72 (0.1)	20 (0.2)	34 (0.2)	0.01
**Clinical characteristics**^**d**^								
**Type of ADHD diagnosis**^e^**, N (%)**								
Inattentive	21,820 (43.9)	8,674 (42.6)	13,146 (44.7)	0.04	10,851 (37.3)	3,348 (35.7)	7,503 (38.1)	0.05
Hyperactive	15,317 (30.8)	6,646 (32.6)	8,671 (29.5)	0.07	13,706 (47.1)	4,639 (49.5)	9,067 (46.0)	0.07
Combined	8,511 (17.1)	3,362 (16.5)	5,149 (17.5)	0.03	2,397 (8.2)	739 (7.9)	1,658 (8.4)	0.02
Other/unspecified	4,107 (8.3)	1,693 (8.3)	2,414 (8.2)	0.00	2,137 (7.3)	651 (6.9)	1,486 (7.5)	0.02
Unknown	1 (0.0)	0 (0.0)	1 (0.0)	0.01	2 (0.0)	0 (0.0)	2 (0.0)	0.01
**Comorbidities associated with ADHD **^**f**,**g**^**, N (%)**								
Acute upper respiratory infections	13,105 (26.3)	5,144 (25.2)	7,961 (27.1)	0.04	6,613 (22.7)	2,103 (22.4)	4,510 (22.9)	0.01
Acute pharyngitis	7,701 (15.5)	3,071 (15.1)	4,630 (15.8)	0.02	3,746 (12.9)	1,213 (12.9)	2,533 (12.8)	0.00
Depression	5,513 (11.1)	2,062 (10.1)	3,451 (11.7)	0.05	6,496 (22.3)	1,789 (19.1)	4,707 (23.9)	0.12
Anxiety disorders	5,473 (11.0)	1,853 (9.1)	3,620 (12.3)	0.10	5,161 (17.7)	722 (7.7)	1,498 (7.6)	0.00
**Prior use of other pharmacological treatments**								
Penicillins	8,465 (17.0)	3,349 (16.4)	5,116 (17.4)	0.03	3,605 (12.4)	1,184 (12.6)	2,421 (12.3)	0.01
Antiasthmatic and bronchodilator agents	5,959 (12.0)	2,356 (11.6)	3,603 (12.3)	0.02	2,801 (9.6)	905 (9.7)	1,896 (9.6)	0.00
Dermatologicals	3,967 (8.0)	1,546 (7.6)	2,421 (8.2)	0.02	4,532 (15.6)	1,427 (15.2)	3,105 (15.7)	0.01
Cephalosporins	3,801 (7.6)	1,486 (7.3)	2,315 (7.9)	0.02	1,795 (6.2)	555 (5.9)	1,240 (6.3)	0.02
Macrolides	3,520 (7.1)	1,341 (6.6)	2,179 (7.4)	0.03	2,360 (8.1)	745 (7.9)	1,615 (8.2)	0.01
Nasal agents—systemic and topical	2,902 (5.8)	1,143 (5.6)	1,759 (6.0)	0.02	1,599 (5.5)	536 (5.7)	1,063 (5.4)	0.01
Corticosteroids	2,832 (5.7)	1,148 (5.6)	1,684 (5.7)	0.00	1,770 (6.1)	546 (5.8)	1,224 (6.2)	0.02
Antidepressants	2,761 (5.5)	878 (4.3)	1,883 (6.4)	0.09	4,829 (16.6)	1,426 (15.2)	3,403 (17.3)	0.06

*ADHD *Attention-deficit/hyperactivity disorder,* SD *Standard deviation

^a^ Patient characteristics were reported for all patients included in the study. Results were stratified by children and adolescents with ADHD and by treatment change cohort (i.e., no treatment change cohort, including patients who did not have a treatment change observed during the 12-month study period [i.e., remained on the first treatment regimen observed]; and treatment change cohort, including patients who had ≥ 1 treatment change observed during the 12-month study period)

^b^ The absolute standardized difference is reported as the difference between the no treatment change cohort and treatment change cohort. A standardized difference of greater than 0.1 was considered to be an important difference

^c^ Demographic characteristics were measured as of the index date, defined as the first observed ADHD-related treatment that was preceded by 6 months of no ADHD-related treatments observed in pharmacy claims. Characteristics were reported for the first 3 treatment regimens of selected ADHD-related agents observed in pharmacy claims. Treatment sequences of treatment regimens observed during the 12-month study period (i.e., the 12-month period following the index date) were defined as all ADHD-related agents observed within 30 days of the first ADHD-related agent and were identified using prescription fills during the 18-month follow-up period (i.e., the 18-month period following the index date)

^d^ Clinical characteristics were reported during the baseline period, defined as the 6-month period prior to the index date. The index date was defined as the first observed ADHD-related treatment that was preceded by 6 months of no ADHD-related treatments observed in pharmacy claims

^e^ The prevalence of combined ADHD was likely an underestimation as data on the diagnosis of combined ADHD was only available from October 2015 when ICD-10-CM codes were implemented

^f^ Based on Kessler, R. C., Adler, L., Barkley, R., Biederman, J., Conners, C. K., Demler, O., & Spencer, T. (2006). The prevalence and correlates of adult ADHD in the United States: Results from the National Comorbidity Survey Replication. American Journal of psychiatry, 163(4), 716–723

^g^ Based on American Psychiatric Pub (2013). Diagnostic and Statistical Manual of Mental Disorders (DSM-5®) and Quan, H., Sundararajan, V., Halfon, P., Fong, A., Burnand, B., Luthi, J. C & Ghali, W. A. (2005). Coding algorithms for defining comorbidities in ICD-9-CM and ICD-10 administrative data. Medical care, 43(11), 1130–1139

A total of 29,093 adolescents with ADHD who met the study criteria were included. Among adolescents with ADHD, the mean age was 15.0 years and 38.1% were female; these demographics were similar to that of the sample prior to requiring continuous eligibility. The first observed ADHD diagnosis among adolescents was commonly inattentive ADHD (47.1%), followed by hyperactive ADHD (37.3%), and combined type ADHD (8.2%). During the 6-month baseline period, 22.3% of adolescents with ADHD had depression and 17.7% had anxiety disorders. Other common baseline comorbidities included acute upper respiratory infections (22.7%) and acute pharyngitis (12.9%). Frequently used pharmacological treatments during the baseline period among adolescents with ADHD included antidepressants (16.6%), dermatologicals (15.6%), and penicillins (12.4%) (Table 1).

### Treatment characteristics of the first regimen observed

The majority (91.9%) of children with ADHD initiated a stimulant in the first treatment regimen observed, with 82.1% initiating a long-acting stimulant and 14.5% initiating a short-acting stimulant. Methylphenidate-based agents were the most common long-acting stimulants (60.9%) and short-acting stimulants (67.2%) received. Non-stimulants were initiated by 9.7% of children, with guanfacine being the most common non-stimulant received (51.8%). As the first treatment regimen observed, 10.5% of children received a combination therapy (i.e., ≥ 2 ADHD-related agents). The mean duration of the first treatment regimen observed among children with ADHD was 7.2 months (Table 2). Throughout the duration of the treatment regimen, 28.2% of children also received psychotherapy, with an average of 6.6 visits.

**Table 2:** Treatment characteristics among children and adolescents with ADHD – First treatment regimen observed

Treatment characteristics	First treatment regimen observed^a^	
	**Children**	**Adolescents**
	***N* = 49,756**	***N* = 29,093**
**Type of pharmacological treatment**^**b**^**, N (%)**		
**Stimulants**	45,740 (91.9)	26,886 (92.4)
**Stimulants – long-acting**	40,867 (82.1)	24,118 (82.9)
**Methylphenidate-based ‒ long-acting**	24,894 (50.0)	10,991 (37.8)
Methylphenidate	18,920 (38.0)	9,097 (31.3)
Dexmethylphenidate	6,293 (12.6)	1,959 (6.7)
**Amphetamine-based ‒ long-acting**	17,579 (35.3)	13,777 (47.4)
Mixed amphetamine salts (i.e., amphetamine + dextroamphetamine)	8,184 (16.4)	5,864 (20.2)
Lisdexamfetamine dimesylate	9,347 (18.8)	8,022 (27.6)
Dextroamphetamine	104 (0.2)	66 (0.2)
Amphetamine	221 (0.4)	36 (0.1)
**Stimulants—short-acting**	7,214 (14.5)	4,657 (16.0)
**Methylphenidate-based ‒ short-acting**	4,851 (9.7)	1,934 (6.6)
Methylphenidate	3,824 (7.7)	1,471 (5.1)
Dexmethylphenidate	1,043 (2.1)	464 (1.6)
**Amphetamine-based ‒ short-acting**	2,474 (5.0)	2,759 (9.5)
Mixed amphetamine salts (i.e., amphetamine + dextroamphetamine)	2,292 (4.6)	2,622 (9.0)
Dextroamphetamine	112 (0.2)	76 (0.3)
Amphetamine sulfate	76 (0.2)	65 (0.2)
**Non-stimulants**	4,808 (9.7)	2,563 (8.8)
Guanfacine	2,492 (5.0)	909 (3.1)
Atomoxetine	2,133 (4.3)	1,595 (5.5)
Clonidine	236 (0.5)	83 (0.3)
**Treatment combination of ≥ 2 therapeutic agents, N (%)**	5,230 (10.5)	3,063 (10.5)
**Psychotherapy, N (%)**	14,052 (28.2)	10,425 (35.8)
**Treatment regimen duration (months)**^**c**^**, mean ± SD [median]**	7.16 ± 4.80 [7.57]	6.45 ± 4.69 [5.60]

*ADHD *Attention-deficit/hyperactivity disorder*, SD *Standard deviation

^a^ Treatment characteristics were reported for the first treatment regimen observed of selected ADHD-related agents observed in pharmacy claims

^b^ Treatment regimen can consist of multiple ADHD-related agents of varying pharmacological types (categories are not mutually exclusive)

^c^ Treatment regimen duration was defined as the time period between the start of the treatment regimen and the end of the treatment regimen. The start of a treatment regimen was defined as the date of the first prescription fill of an ADHD-related agent. The end of a treatment regimen was defined as the date of the first occurrence between (1) the first ADHD-related treatment change (i.e., treatment discontinuation, treatment switch, treatment add-on, treatment drop) and (2) the end of the 12-month study period

The majority (92.4%) of adolescents with ADHD initiated a stimulant, with 82.9% initiating a long-acting stimulant and 16.0% initiating a short-acting stimulant. Amphetamine-based agents were the most common long-acting stimulants (57.1%) and the most common short-acting stimulants (59.2%) received. Non-stimulants were initiated by 8.8% of adolescents, with atomoxetine being the most common non-stimulant received (62.2%). As the first treatment regimen observed, 10.5% of adolescents received a combination therapy (i.e., ≥ 2 ADHD-related agents). The mean duration of the first treatment regimen observed among adolescents with ADHD was 6.5 months (Table 2). Throughout the duration of the treatment regimen, 35.8% of adolescents also received psychotherapy, with an average of 6.9 visits.

### Treatment changes

**Fig. 2:**
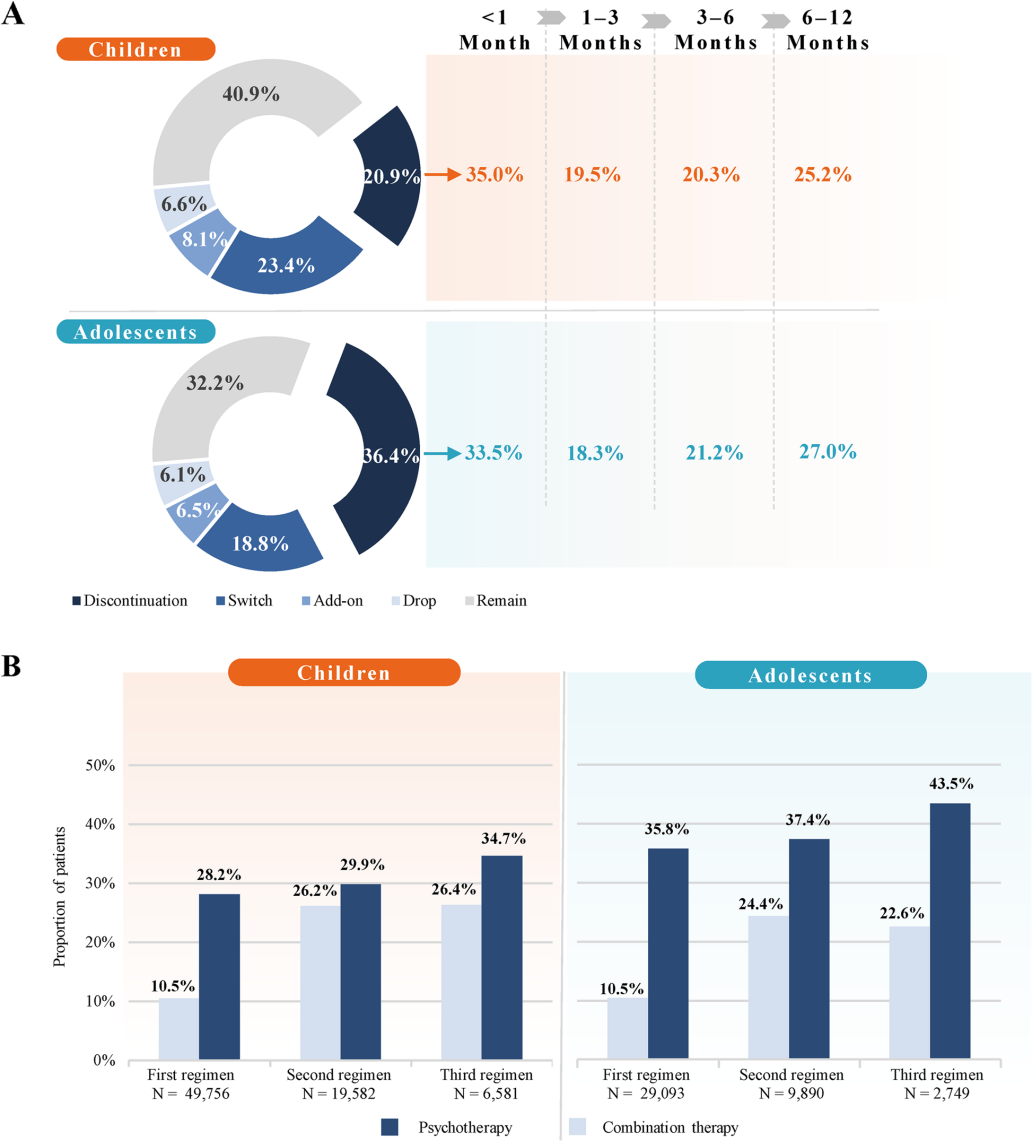
Treatment changes and psychotherapy and combination therapy trends. **A** Pharmacological treatment changes at the end of the first regimen observed and time to discontinuation^1−4^. **B** Combination and psychotherapy use trend^5^. Notes: [1] Treatment discontinuation was defined as having no ADHD-related agents (of any type) for at least 180 consecutive days following the last day of supply of all ADHD-related agents included in the previous treatment regimen. [2] Treatment switch was defined as the initiation of a new ADHD-related agent (not part of the previous treatment regimen) with no prescription fills of the last ADHD-related agent(s) included in the previous treatment regimen within the 30 days following the newly initiated ADHD-related agent. [3] Treatment add-on was defined as the initiation of a new ADHD-related agent (not part of the previous treatment regimen) with at least one additional prescription fill of the last ADHD-related agent(s) included in the previous treatment regimen, within the 30 days following the newly initiated ADHD-related agent. [4] Treatment drop was defined as the discontinuation of an ADHD-related agent from a treatment regimen while the other agent(s) from treatment regimen are not discontinued for at least enough time to define a new regimen. [5] Psychotherapy and combination therapy of ≥ 2 therapeutic agents were measured between the start and the end of the treatment regimen. Psychotherapy may be underreported in claims

At the end of the 12-month study period, 40.9% of children with ADHD had remained on their first treatment regimen observed and 59.1% had experienced a treatment change. Among children who experienced a treatment change on their first regimen observed, 20.9% had a treatment discontinuation, 23.4% had a switch, 8.1% had an add-on, and 6.6% had a drop. For those who discontinued treatment, discontinuation occurred within the first month following the initiation of the first treatment regimen observed in 35.0% of patients (Fig. 2A). Over the 12-month study period, 3,285 unique treatment sequences at the agent level were observed among children with ADHD, with 37.8% of children experiencing 1 treatment change, 15.4% experiencing 2 treatment changes, and 5.8% experiencing ≥ 3 treatment changes. Additionally, increasing proportions of combination therapy and psychotherapy use were observed in subsequent regimens (Fig. 2B).

At the end of the 12-month study period, 32.2% of adolescents with ADHD had remained on their first treatment regimen observed and 67.8% had experienced a treatment change. Among adolescents who experienced a treatment change on their first regimen observed, 36.4% had a treatment discontinuation, 18.8% had a switch, 6.5% had an add-on, and 6.1% had a drop. For those who discontinued treatment, discontinuation occurred within the first month following the initiation of the first treatment regimen observed in 33.5% of patients (Fig. 2A). Over the 12-month study period, 1,733 unique treatment sequences at the agent level were observed among adolescents with ADHD, with 47.5% of adolescents experiencing 1 treatment change, 15.9% experiencing 2 treatment changes, and 4.4% experiencing ≥ 3 treatment changes. Additionally, increasing proportions of combination therapy and psychotherapy use were observed in subsequent regimens (Fig. 2B).

**Fig. 3:**
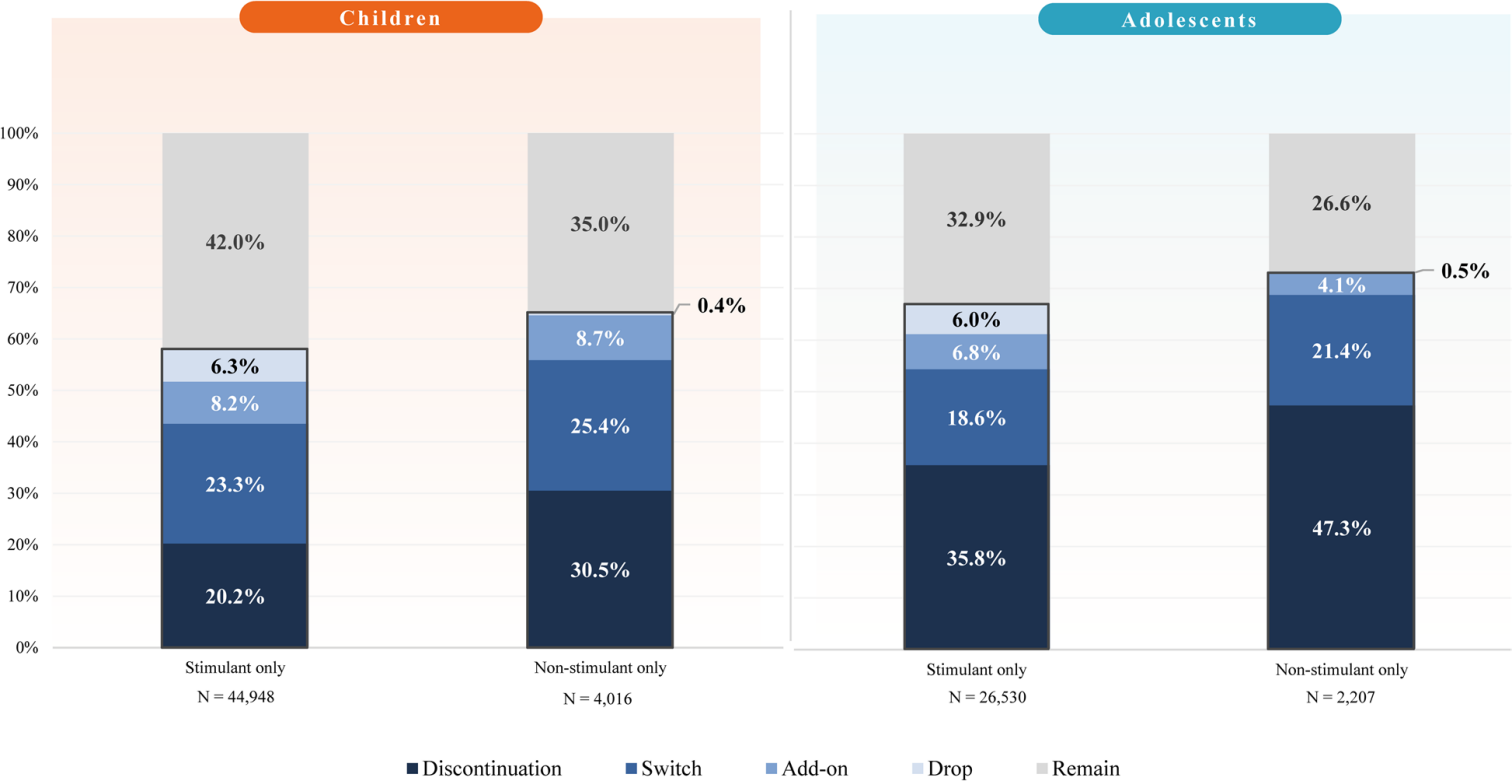
Treatment changes by treatment class of first regimen received^1−5^. Notes: [1] Treatment discontinuation was defined as having no ADHD-related agents (of any type) for at least 180 consecutive days following the last day of supply of all ADHD-related agents included in the previous treatment regimen. [2] Treatment switch was defined as the initiation of a new ADHD-related agent (not part of the previous treatment regimen) with no prescription fills of the last ADHD-related agent(s) included in the previous treatment regimen within the 30 days following the newly initiated ADHD-related agent. [3] Treatment add-on was defined as the initiation of a new ADHD-related agent (not part of the previous treatment regimen) with at least one additional prescription fill of the last ADHD-related agent(s) included in the previous treatment regimen, within the 30 days following the newly initiated ADHD-related agent. [4] Treatment drop was defined as the discontinuation of an ADHD-related agent from a treatment regimen while the other agent(s) from treatment regimen are not discontinued for at least enough time to define a new regimen. [5] Patients who received a stimulant and a non-stimulant in combination therapy (1.6% of children, 1.2% of adolescents) were excluded

When stratified by the treatment class received, among children with ADHD whose first treatment regimen observed consisted of only stimulants, 58.0% experienced a treatment change (20.2% treatment discontinuation; 23.3% treatment switch; 8.2% treatment add-on; 6.3% treatment drop), and among those with only non-stimulants, 65.0% experienced a treatment change (30.5% treatment discontinuation; 25.4% treatment switch; 8.7% treatment add-on; 0.4% treatment drop; Fig. 3). Among adolescents with ADHD whose first treatment regimen observed consisted of only stimulants, 67.1% experienced a treatment change (35.8% treatment discontinuation; 18.6% treatment switch; 6.8% treatment add-on; 6.0% treatment drop), and among those with only non-stimulants, 73.4% experienced a treatment change (47.3% treatment discontinuation; 21.4% treatment switch; 4.1% treatment add-on; 0.5% treatment drop; Fig. 3).

### Healthcare costs

**Fig. 4:**
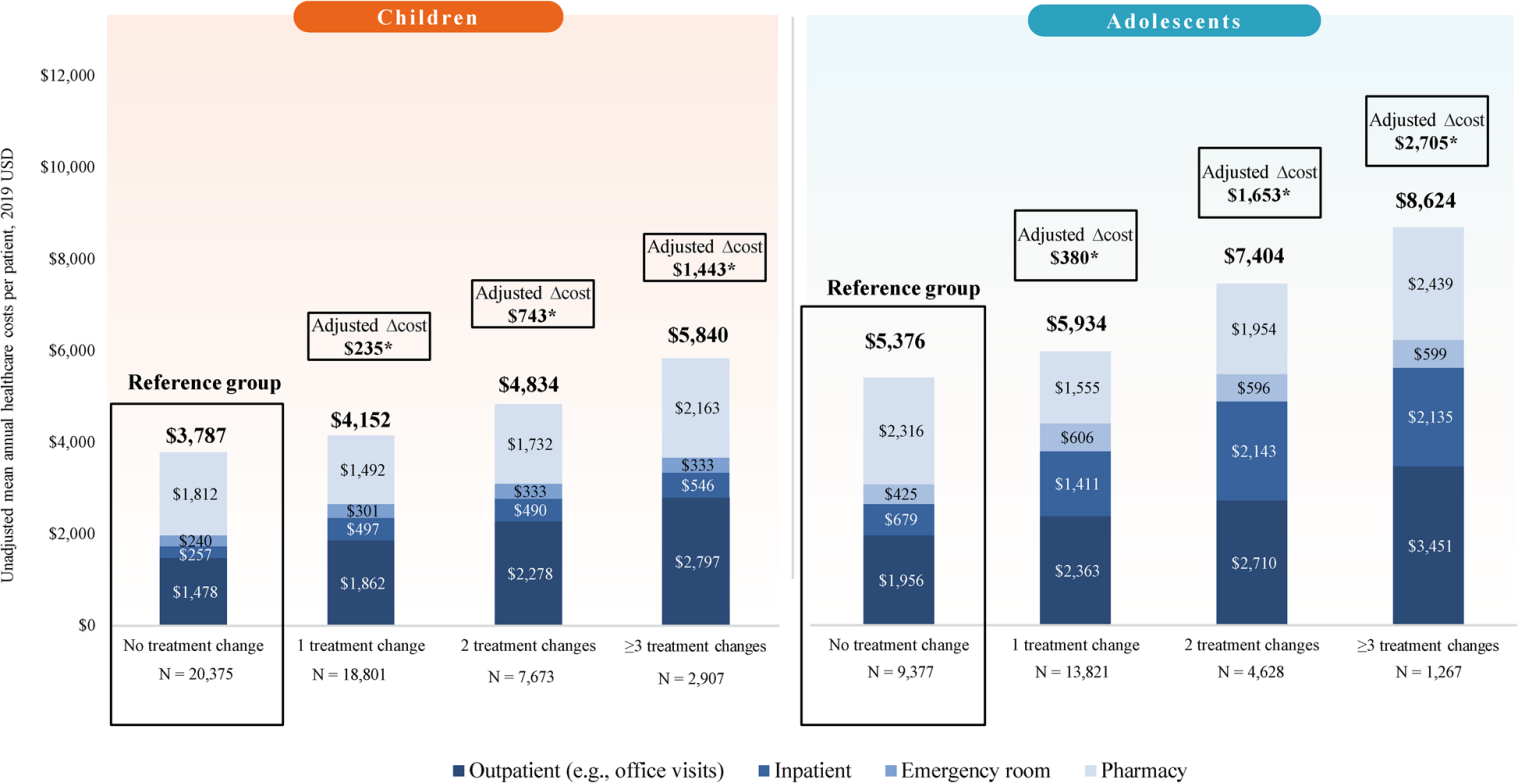
Unadjusted mean annual healthcare costs per patient per year and adjusted cost differences.^1,2^. * Significant at the 5% level. Notes: [1] Total healthcare costs per patient incurred during the 12-month study period were calculated among patients who did not experience a treatment change and among those who experienced 1, 2, or ≥ 3 treatment changes. Total healthcare costs were calculated from the payer perspective and adjusted to 2019 USD using the US Medical Care Consumer Price Index. [2] The unadjusted and adjusted cost differences for each cost component were estimated using generalized linear regression models. The adjusted models were adjusted for the following demographic and baseline characteristics in which the standardized difference between those with and without a treatment change was ≥ 0.1: age, gender, region, health plan, type of ADHD diagnosis, year of index date, anxiety (children only), patients with ≥ 1 psychotherapy visit (children only), depression (adolescents only)

The unadjusted total annual healthcare costs per child with ADHD who did not experience a treatment change over the 12-month study period was $3,787. Adjusted regression models estimated that children with 1, 2, and ≥ 3 treatment changes over the 12-month study period incurred excess annual healthcare costs of $235 (95% CI: $10, $460), $743 (95% CI: $414, $1,071), and $1,443 (95% CI: $1,060, $1,826) (all *p* < 0.05), respectively, which were mainly driven by excess medical costs, particularly by outpatient costs (e.g., office visits; Fig. 4). Therefore, a positive relationship was observed between the number of treatment changes and total adjusted healthcare cost differences, with children with ADHD who had 1, 2, and ≥ 3 treatment changes incurring an additional 6.2, 19.6, and 38.1% in adjusted annual healthcare costs relative to those without a treatment change, respectively (Fig. 4).

The unadjusted total annual healthcare costs per adolescent with ADHD who did not experience a treatment change over the 12-month study period was $5,376. Adjusted regression models estimated that adolescents with 1, 2, and ≥ 3 treatment changes over the 12-month study period incurred excess annual healthcare costs of $380 (95% CI: $0, $761), $1,653 (95% CI: $562, $2,744), and $2,705 (95% CI: $1,593, $3,816) (all *p* ≤ 0.05), respectively, which were mainly driven by excess medical costs, particularly by inpatient costs (Fig. 4). Adolescents with ADHD who had 1, 2, and ≥ 3 treatment changes incurred an additional 7.1, 30.7, and 50.3% in adjusted annual healthcare costs relative to those without a treatment change, respectively (Fig. 4).

## Discussion

This large real-world analysis demonstrated that a sizable portion of children and adolescents with ADHD experienced a treatment change within 12 months of initiating a new ADHD treatment. In both populations, most patients initiated a stimulant, with increasing use of combination therapy and psychotherapy as patients progressed to subsequent treatment regimens. Treatment discontinuation and switch were particularly common; among those who discontinued, over one-third of patients did so within the first month of treatment. Overall, the 3,285 and 1,733 distinct treatment journeys observed within a 12-month period among children and adolescents, respectively, highlight that there appears to be no clear treatment path and patients with ADHD cycle through various treatment options. In addition to the patient burden associated with such treatment cycling, the frequent treatment changes observed was associated with excess healthcare costs, which increased as patients experienced additional treatment changes. Collectively, the findings of this study suggest that the current treatment options may not sufficiently meet the needs of children and adolescents with ADHD.

Previous studies evaluating treatment patterns in children and adolescents with ADHD have focused on specific drugs or drug classes [18–20, 27], and most cost analyses in ADHD have not evaluated healthcare costs associated with treatment changes [20, 21]. The current study expands the literature by capturing multiple treatment changes/sequences and the associated healthcare costs in these populations. Findings of the current study corroborated previous reports on the high rates of treatment discontinuation and switch that frequently occur within just a few months of treatment. A novelty of the current study is the use of a specific algorithm to capture various types of treatment changes and to stratify treatment patterns by class of treatment regimen received, which allowed for a comprehensive assessment of the respective treatment journey of children and adolescents with ADHD. Additionally, the definition of treatment discontinuation of ≥ 180 days is an important differentiation from previous studies, which generally used smaller treatment gaps when defining discontinuation [15]. Since it is common for children and adolescents with ADHD to omit medications during non-school days, when demand on attention is less, for reasons such as to reduce the experience of adverse effects from the medications [28, 29], the use of smaller gaps may capture these transient treatment interruptions, thereby overestimating discontinuation. Nonetheless, despite a conservative definition used in the current study, discontinuation was still commonly observed. Meanwhile, the use of data from a large claims database in the current study also allowed for the assessment of the association between healthcare costs and multiple treatment changes that has not been previously reported among children and adolescents with ADHD.

The findings that a high proportion of children and adolescents with ADHD underwent treatment changes during a 12-month period suggest that many patients continue to search for more optimal treatments after receiving the initial regimens. Literature evidence suggests that reasons for treatment changes could be treatment-related (e.g., adverse effects, suboptimal response, risk of abuse) [15], which implies advancement in treatment options may help reduce the rates of treatment changes. Understandably, treatment response may vary across individuals and a treatment change may not always indicate ineffective medication (e.g., stepped-care approaches [30]). It is of note that a stepped-care approach would mainly be captured as treatment add-on, which constituted a relatively small portion of treatment changes observed in this study. Meanwhile, a treatment change may be due to the emergence of new health problems or complications resulting from existing comorbidities [31], prompting the need for a new treatment plan. Alternatively, a treatment change may be part of a treatment plan that aims to better control comorbid symptoms [32]. Additionally, treatment changes may also be triggered by patient-related factors (e.g., lack of understanding of medication need, parents’ opinion), access-related (e.g., pharmacy shortages) factors [15, 33, 34], or cost factors (e.g., discontinue a treatment because it is no longer affordable or switch to a cheaper alternative). Importantly, the rationale for a treatment change may be patient-specific but may also be population-specific and differ between children and adolescents with ADHD, as suggested by the discrepancies in treatment patterns between these populations observed in this study. For example, children with ADHD appeared to experience more treatment switches than adolescents, whereas adolescents appeared to experience more treatment discontinuation and more treatment changes overall than children, highlighting the potential differences in ADHD management faced by these populations. Indeed, patient age has been shown to be a predictor of ADHD treatment discontinuation among children and adolescents, with higher risks of discontinuation and shorter time on therapy among patients ≥ 12 years compared to those < 12 years [35, 36]. The increased rate of treatment changes observed among adolescents with ADHD may be due to the patients’ evolving social roles with age, including increased responsibilities, autonomy in managing treatment schedules, and mounting peer pressure [4, 7], all of which may present as barriers to treatment adherence and persistence among adolescents. Additionally, a higher proportion of adolescents in the current cohort had inattentive ADHD than hyperactive ADHD, which was consistent with previous reports [3, 37, 38]. The characteristics of inattentive ADHD (e.g., difficulty with organization, forgetfulness [4]) may also contribute to poor persistence among adolescents. Future investigations are warranted to better understand the reasons underlying different types of treatment changes and associated reasons for change among children and adolescents with ADHD.

In addition to the direct patient burden of cycling through treatment and incomplete ADHD symptom management, this study also revealed the increased healthcare costs associated with these treatment changes, which may have important implications. ADHD has been found to be a costly condition to society [39, 40], with an estimated $9.0 billion annually being attributable to direct healthcare costs among children and adolescents with ADHD in the US [40]. The findings of the current study demonstrate that more treatment changes are associated with higher healthcare costs, which may be contributing to the large societal burden associated with direct healthcare costs among patients with ADHD. Clinicians and other stakeholders such as payers may be more prudent on treatment decisions and recommendations by recognizing the increased costs that come with a treatment change, and ultimately the resulting impact that this may have on society. Of note, the overall costs associated with treatment changes found in this study may be underestimated, as there are likely considerable indirect costs, including education-related costs (e.g., missed school days) and spillover costs to families and caregivers (e.g., productivity loss due to the need for additional medical attention and physician visits).

The findings of this study should be considered in light of limitations. This study was conducted in a commercially insured population, and hence results may not be representative of the general ADHD population or individuals with public or no health insurance. The algorithm used to define treatment sequences was based solely on the timing of claims with ADHD-related agents, as clinical information is unavailable in claims data. As with all claims-based studies, this study is also subject to general limitations such as billing inaccuracies and missing data. Furthermore, the potential impact of unobservable variables on the study results could not be measured. Additionally, since patients included in the study were captured along different trajectories of their treatment journey, it was not possible to differentiate whether the first observed treatment was the first treatment after the diagnosis of ADHD. As patient’s treatment journey may not be captured in its entirety, the results should be interpreted in view of the inclusion of a mixed population of patients with different treatment history that may affect clinical decisions.

## Conclusions

Over a 1-year period, treatment changes were common among children and adolescents with ADHD in the US and the number of treatment changes was positively associated with excess total annual healthcare costs, suggesting potential unmet treatment needs within these populations. Programs, interventions, and treatment options that offer additional solutions to manage patients ADHD symptoms and improve treatment persistence may result in improved treatment outcomes and reduced overall burden to patients and society.

## Data Availability

The datasets generated and analyzed during the current study are not publicly available because they were used pursuant to a data use agreement. Requests for data should be made directly to IBM.

## Abbreviations

ADHD: Attention-deficit/hyperactivity disorder
CI: Confidence interval
FDA: Food and Drug Administration
US: United States
